# Prevalence of ST1193 clone and IncI1/ST16 plasmid in *E-coli* isolates carrying *bla*_CTX-M-55_ gene from urinary tract infections patients in China

**DOI:** 10.1038/srep44866

**Published:** 2017-03-24

**Authors:** Liang Xia, Yang Liu, Shu Xia, Timothy Kudinha, Shu-nian Xiao, Nan-shan Zhong, Guo-sheng Ren, Chao Zhuo

**Affiliations:** 1 State Key Laboratory of Respiratory Disease, First Affiliated Hospital of Guangzhou Medical University, Guangzhou 510230, China; 2 The First Affiliated Hospital of Chongqing Medical University, Chongqing 400016, China; 3 Charles Sturt University, Leeds Parade, Orange, New South Wales, Australia

## Abstract

To study molecular epidemiology of CTX-M-55-carrying *Escherichia coli* isolates from urinary tract infections (UTIs) in China. 111 *bla*_CTX-M-55_-positive *E.coli* isolates from UTIs patients in China were studied. Pulsed-field gel electrophoresis (PFGE) and multilocus sequence typing (MLST) were used to analyze the homologies among the strains. Conjugation experiments, S1nuclease PFGE and PCR analysis were performed to characterize plasmids harboring *bla*_CTX-M-55_ and their genetic environment. 111 isolates were clustered into 86 individual pulsotypes and three clusters by PFGE. Fifty-five (49.5%) of the isolates belonged to 8 STs. Most of the ST1193 isolates belonged to one PFGE cluster. Transconjugants (n = 45) derived from randomly selected *bla*_CTX-M-55_ donors (n = 58), were found to contain a single 90-kb conjugative plasmid, which mainly belonged to the IncI1 groups (34, 76%). Among the IncI1 plasmids, the *bla*_CTX-M-55_/IncI1/ST16 predominated (23/34, 68%). The *bla*_TEM-1_ and *aac (3*′)*-II* genes were frequently detected on the IncI1 plasmids, and the insertion of IS*Ecp1* or IS*26* was observed at the 48 bp or 45 bp upstream of the start codon of *bla*_CTX-M-55_ gene. The dissemination of *bla*_CTX-M-55_ gene among *E. coli* UTI isolates, appeared to be due to both the major clonal lineage of ST1193 and the horizontal transfer of epidemic plasmid IncI1/ST16.

Urinary tract infections (UTIs) are the most common bacterial infection and have a significant economic impact on the health care system. About 70–95% of community-acquired, and 50% of nosocomial UTIs are caused by *E. coli*^1^. There is increasing evidence that epidemic clonal groups of *E. coli* are responsible for a rise in antimicrobial-resistance in UTIs. For example, the prevalence of extended spectrum beta lactamase (ESBL)-producing *E. coli* (ESBL-EC) sequence type (ST131) is increasing worldwide, accounting for 25% and 54% of all ESBLs in Mexico and the United States, respectively^2^,^3^. Moreover, the emergence of drug-resistant epidemic plasmids, that confer antimicrobial resistance among bacteria, also contributes to increased resistance, through the horizontal transmission of ESBL-encoding genes. IncFII, IncA/C, IncL/M, IncN and IncI1 plasmids, carrying *bla*_CTX-M_ genes and acquired *ampC* genes, are frequently detected in *Enterobacteriaceae* of different origin and source^4^. Specifically, the IncFII-like type plasmids have been reported to be closely associated with *E. coli* ST131 carrying *bla*_CTX-M-15_, while the IncI1 plasmids carrying *bla*_CTX-M-15_ or *bla*_CMY-2_ are frequently isolated from non-ST131 *E. coli* strains, some of which are shared between humans and animals^5^,^6^.

In China, ST131 was the most abundant type (12.7%) in 256 ESBL-producer isolates based on a study on ESBL-EC isolates from patients with community-onset infections in Chinese country hospitals from 2010 to 2011^7^, and its prevalence increased to 29.5% in another study on bloodstream infections patients in three hospitals in China from 2013 to 2014^8^. However, *E. coli* isolates from UTIs show a significantly different drug resistant profile than those from Europe, with more than 50% of isolates from China estimated to be resistant to both ciprofloxacin and cefotaxime by the year 2000. Since 1998, the predominant ESBL type in *E. coli* in China has been CTX-M-14, which was detected in 71% of the ESBL-ECs in 2008^9^,^10^. However, a shift in ESBL genotypes has been reported recently, with the prevalence of CTX-M-15 and CTX-M-55, which are members of the CTX-M-1 group, increasing significantly in China since 2011^11^. According to the national surveillance program in China, the *bla*_CTX-M-15_ positive strains accounted for 24% of all ESBL-EC UTI isolates that harbored *bla*_CTX-M_ genes in 2011. But as of 2013,the situation had changed, with *bla*_CTX-M-15_ and *bla*_CTX-M-55_ positive strains accounting for 15% and 21% of all ESBL-EC UTI isolates, respectively^10^,^11^. Interestingly, the emerging *bla*_CTX-M-55_ ESBL genotype was also detected in *E. coli* strains from food animals or pets, according to different survey programs conducted from 2006–2012^12^,^13^. These findings suggest that the high prevalence of *bla*_CTX-M-55_ in animals might contribute to the spread of *bla*_CTX-M-55_ genes between animals and humans. Unfortunately, there is limited data to clarify the clonal relationship between these *bla*_CTX-M-55_-carrying *E. coli* isolates from humans and animals. Furthermore, to our knowledge, compared to several molecular studies on *bla*_CTX-M-55_ in ESBL-EC strains of animal origin, there are limited studies on *bla*_CTX-M-55_ from clinical ESBL-EC strains from humans both in China and worldwide.

Consequently, in the present study, we examined the occurrence and characteristics of *bla*_CTX-M-55_ carrying ESBL-EC from UTIs following our previous study^11^. The plasmids harbored by these isolates, as well as the association between *bla*_CTX-M-55_ and other resistance genes, were also investigated.

## Results

### Distribution of isolates in demographics and source

In this study, a total of 111 CTX-M-55-producing *E. coli* isolates were obtained from urine specimens from 21 general hospitals in 19 provinces in China. These included 29 isolates from the Eastern (26%), 6 from the Southern (5%), 26 from the Northern (23%), 11 from the Central (10%), 12 from the Northeast (11%), 12 from the Southwest (11%), and 15 from the Northwest (13%) regions (data not shown). The majority of the isolates, 62(56%) were from hospital acquired UTIs, and the rest from community onset infection. Most of the isolates, 87(78%) were from females, and the median age was 60 years (range, 10 months to 91years).

### Antimicrobial susceptibility patterns

All the 111 CTX-M-55- producing *E. coli* isolates were highly susceptible to imipenem, meropenem,ertapenem, piperacillin/tazobactam, tigecycline, and colistin, at approximately 93–100%. However, 76–98% of the isolates were resistant to ciprofloxacin, levofloxacin, cefotaxime, cefepime, ceftazidime, aztreonam, and ceftriaxone (Table S1).

### PFGE profiles

Using PFGE, the CTX-M-55-producing *E. coli* isolates were discriminated into 89 pulsotypes (figure not shown), composed of 86 individual pulsotypes, and three clusters (I, II, III) for the remaining 25 isolates. Cluster I, which was defined at the 80% similarity level, encompassed 20(18%) isolates, and covered 14 hospitals in all the seven geographical regions across China (Figure S1). Cluster II and cluster III encompassed two and three isolates each, respectively.

### MLST results

The CTX-M-55-producing *E. coli* isolates were grouped into 48 distinct STs, with the predominant ST being ST1193 (n = 20, 18%), followed by ST405 (n = 9, 8.1%), ST156 and ST2003 (5, 4.5% each), ST12, ST95, ST167, and ST354 (4, 3.6% each). These eight major STs accounted for 49.5% of the studied isolates (Table S2). A further 4 STs (ST10, ST 38, ST101, ST131) comprised of 3 isolates each, whilst the rest (36 STs) occurred in one or 2 isolates each. Three STs, including ST4207, ST4208 and ST4210, were novel to this study. An overwhelming majority of ST1193 isolates (18 of 20) were classified into the cluster I pulsotype by PFGE, whilst the isolates from other STs were scattered in diverse pulsotypes. Greater diversity was observed in isolates from hospital acquired UTIs, belonging to 35 different STs, than among isolates from community acquired UTIs, which belonged to 23 STs.

eBURST analysis revealed one clonal complex CC(CC10) encompassing 8 STs including ST10, ST167, ST44, ST744, ST1634, ST1488, ST617, and ST4204, which represented 13 isolates, whereas the remaining 40 profiles were observed in a single isolate each (Figure S2). Interestingly, ST1193(and other 38) were not assigned to a clonal complex, even when less stringent conditions were employed (i.e. STs sharing four or more alleles; data not shown). We further examined the clonality of our STs in an eBURST comparison with all *E. coli* STs in the international MLST web database (as at April 2014). With this wider comparison, ST1193 appears to belong to a prevalent clonal complex (CC14) (Figure S3). In addition, a novel single-locus variant (SLV) of ST1193, designated ST4207 was identified in one isolate. Furthermore, with regards to the drug susceptibility test results, all ST1193 isolates were susceptible to imipenem, meropenem, ertapenem, tigecycline, colistin and amikacin, and resistant to ciprofloxacin, ceftriaxone, ceftazidime, cefepime, and aztreonam, respectively (data not shown).

### Plasmid analysis

The 20 ST1193 isolates were classified into 12 pulsotypes by PFGE. Among them, 5 isolates originating from one hospital belonged to cluster I, and the remaining 15 isolates belonged to other 11 clusters. So, one out of the five isolates from one hospital, and 15 isolates belonging to other 11 clusters were selected for conjugation studies. Additionally, another 42 randomly selected isolates belonging to other ST types were studied. Successful transfer of plasmids occurred in 77.6% (45 of 58) isolates. The 45 *bla*_CTX-M-55_ donors were the 16 ST1193 strains and the remaining 29 donors were distributed as follows: ST95 (n = 3), ST156 (n = 3), ST167 (n = 3), ST405 (n = 3), ST10 (n = 2), ST131 (n = 2), and 13 other individual ST donors. Plasmid DNA was isolated from 45 transconjugants (Table 1), and subsequent characterization of plasmids revealed that they belonged to the IncI1 plasmid class (n = 34; 76%), the F plasmid family (n = 10; 22%) and IncP (n = 1; 2%). Notably, 88% (14/16) of ST1193 isolates carried IncI1 plasmids (Table 1).

The replicon types of IncF plasmids were FIB (n = 1), FII (n = 1), FIA + FIB (n = 1), FIA + FII (n = 2), FIB + FII (n = 2), FIA + FIB + FI (n = 1) and FIB + FIC + FI (n = 2). IncI1 plasmids were classified into five STs according to the database of plasmid Multi-Locus Sequence Typing (http://pubmlst.org/plasmid/), with IncI1/ST16 the predominant subtype (23/34, 68%), with size of 97 kb. The remainder were ST15 and ST23 (one isolate each), ST136 (n = 2), and 7 were untypable. Of the seven untypable plasmids, a novel combination of alleles (repI1, 1; ardA, 5; trbA, 10; sogS, 8; pilL, 1) was detected in five plasmids, and another novel combination (repI1, 1; ardA, 2; trbA, 8; sogS,4; pilL, 10) was detected in one plasmid, while three alleles of the remaining one plasmid could not be typed. Further typing of the lncF plasmids (10/45, 22%) revealed a variety of subgroups: F18:A-:B1(n = 4,145 kb); F-:A1:B1(n = 1); F-:A-:B20(n = 1); F51:A-:B10(n = 1); and three were untypable.

Characterization of the antibiotic resistance genes and genetic environment of *bla*_CTX-M-55_ revealed a variable genetic context of the IncI1 plasmid. Except for six IncI1 plasmids which contained only *bla*_CTX-M-55_ gene, the remaining plasmids (n = 17) contained at least two other antibiotic resistance genes. The *bla*_TEM-1_ gene and *aac (3*′)*-II* gene were detected in 65% and 44% of IncI1 plasmids, respectively(Table 1). Other antibiotic resistance genes were variably found (data not shown) while the *bla*_SHV_ gene was not detected.

PCR identified the insertion sequence IS*Ecp1* or IS*26* in its entirety or partially truncated upstream of the *bla*_CTX-M-55_ gene in 32 of 45 (71%) transconjugants. Nucleotide sequence analysis of the upstream region of *bla*_CTX-M-55_ genes revealed that the spacer region between the right inverted repeat (IRR) and the start codon of *bla*_CTX-M-55_ genes were 45 bp or 48 bp.

**Figure 1:**
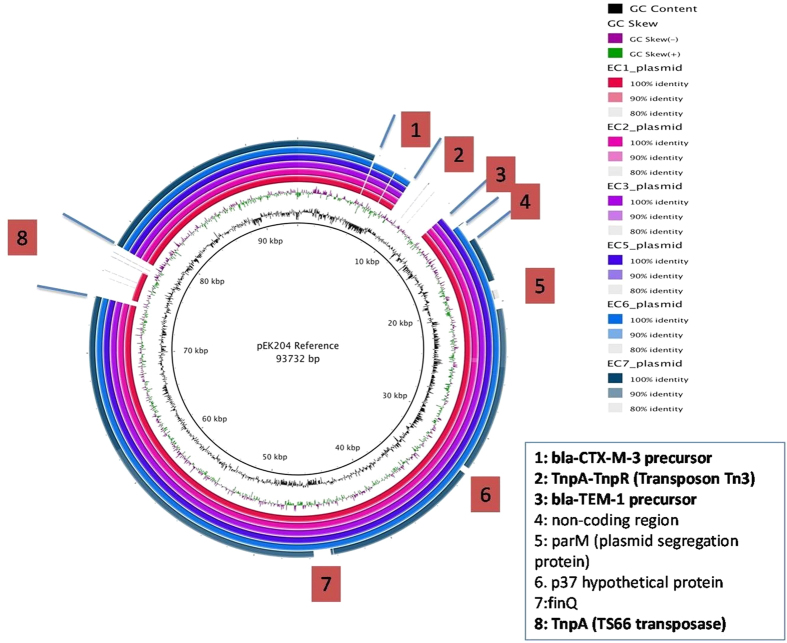
BRIG output image of pseudo-plasmids compared to *E.coli bla*_CTX-15_ plasmid pEK204.

To understand if the IncI1/ST16 type plasmid was a possible epidemic plasmid, six of the IncI1/ST16 plasmids were selected randomly to determine their complete nucleotide sequences. The six plasmids consisted of a large backbone with considerable homology to the pEK204 plasmid isolated from *E. coli* in the UK, except for 8 regions missing in some of the plasmids compared to the reference. As shown in Fig. 1, BRIG output image of pseudo-plasmids compared to *E. coli* bla-CTX-M-15 plasmid pEK204, the innermost rings show GC content (black) and GC skew (purple/green). The remaining rings show BLAST comparisons of the 6 plasmids against the reference plasmid pEK204. The numbers 1–8 highlight the regions missing in some of the plasmids compared to the reference.

## Discussion

The emergence of ESBL-*E.coli* strains carrying *bla*_CTX-M-55_ gene across China poses a great threat to the health care systems, as these strains have been shown to cause UTIs more frequently than those carrying *bla*_CTX-M-15_ gene^11^. Our study provides some evidence that dissemination of the *bla*_CTX-M-55_ in China is partly attributed to the expansion of the *E. coli* ST1193 clone carrying the IncI1/ST16 plasmids. Twenty (18%) of 111 ESBL-EC strains possessing the *bla*_CTX-M-55_ gene and isolated from 24 hospitals among seven regions of China, belonged to a single sequence type (ST), ST1193 and a single PFGE cluster (cluster I). Furthermore, 88% (14 of 16) ST1193 isolates harbored the IncI1 plasmids of the same sequence type, ST16.

Incidentally, ST1193 was the most common *E. coli* ST isolated from both humans and dogs in Australia, and the isolates from both sources had similar serotypes, drug resistance and virulence genes, suggesting transmission between owners and dogs^14^. ST1193 was also prevalent in UTI isolates in Korea^15^. Furthermore, these 2 previous studies showed that ciprofloxacin resistance and lactose non-fermenting are two common phenotypic characteristics of the ST1193 *E.coli*.

Our present work reveals novel features of the ST1193 *E.coli* strains, including carriage of plasmids encoding drug-resistance genes such as *tem-1, aac(3*′) - *II, qnrA* and *arm*, in addition to the *CTX-M-55* gene. We have also previously reported on ST1193 strains possessing the *bla*_CTX-M-15_, *bla*_CTX-M-14_ and *bla*_CMY-2_ genes^11^. Thus the multiple drug resistance due to presence of several antibiotic resistance determinants, might contribute significantly to the successful dissemination of the ST1193 *E.coli* in both the community and hospital environment. On the other hand, pandemic *E. coli* clones such as ST131 often encode multiple virulence gene products associated with mobile genetic elements (MGEs)^16^. It is worth investigating the whole genomic structure of ST1193 in future studies to explain its nationwide dissemination in China.

Besides ST1193, *E. coli* strains from several other STs were also found in about 4% of the isolates in the present study, including ST405(8%), ST156 and ST2003(4.5% each), ST12, ST95, ST167 and ST354(3.6% each). These findings suggest possible dissemination of these clones in China, though on a lower scale than ST1193. Unfortunately, due to cost limitation, we were not able to fully characterize and compare isolates in each of these STs, which would have given further insights. It is highly possible that in future, we will see an increase in the prevalence of these STs as they disseminate further in the community.

Interestingly, there were only three *bla*_CTX-M-55_ carrying ESBL-EC strains in our study which belonged to the international pandemic ST131. This is in sharp contrast to our previous study in 2014, which showed a high percentage of *bla*_CTX-M-14_ carrying UTI *E. coli* ST131^11^. Thus, it is likely that the STs of ESBL-ECs are greatly diversified, perhaps driven by multiple factors, including clinical antibiotic pressure, public health conditions in different regions, and food-borne animal origin.

Plasmids play a key role in horizontal transfer of ESBL genes among *E. coli* strains and thus can significantly contribute to the rise in antibiotic resistance in UTIs. Through typing of ESBL-carrying plasmids, we observed that 76% (34/45) were IncI1 type plasmids, 22% (10/45) Inc F plasmids and 2% (1/45) IncP plasmids, suggesting that these plasmids were contributors to antibiotic resistance and hence dissemination of these strains. IncI1 plasmids belong to narrow-host-range (NHR) type, and carry genes for synthesis of type IV pili, which mediate bacterial adhesion and invasion^17^. The IncI1 plasmids often carry a variety of β-lactamase antibiotic resistant genes such as *bla*_CMY-2_, *bla*_CTX-M-1_ and *bla*_SHV-12_^6^,^18^. Our present findings also show that IncI1 plasmids can also carry resistance determining genes of other antibiotic classes, such as *bla*_TEM-1_, *aac(3*′)*-II, aac-(6*′)-*Ib, qnrA* and *qnrB*, which may contribute to their long-term existence in the intestinal flora of humans and animals, and thus become a reservoir for continual transmission of bacterial strains carrying these genes.

In order to understand the epidemiology of the IncI1 plasmids, Garcia and colleagues established an IncI1 plasmid MLST database (http://pubmlst.org/plasmid/), in which 158 subtypes have been registered. Among these subtypes, the *bla*_CTX-M-1_/IncI1/ST3, *bla*_CTX-M-1_/IncI1/ST7 and *bla*_CMY-2_/IncI1/ST2, are the frequently reported subtypes for ESBL-EC isolates of animal origin (such as poultry and pets) in Europe and America^6^,^18^,^19^. IncI1 plasmids carrying the *bla*_CTX-M-55_ were reported in ESBL-EC isolates of animal origin (dogs, chickens, ducks, etc.) from surveillance studies in Southern China, but unfortunately the MLST subtypes of these plasmids remain unknown^13^.

Contrary to these previous studies, the IncI1/ST16 subtype was the predominant subtype in the present study, constituting 68% of IncI1 carrying the *bla*_CTX-M-55_ gene.

We also identified another five IncI1/ST16 closely related plasmids that might be IncI1/ST16 variants due to one single allele gene mutation (pilL6 → 1), compared to the sequence of IncI1/ST16. The IncI1/ST16 subtype was detected first in the British bovine *E. coli* isolate (pEK204), and was subsequently reported in a French dog *E. coli* isolate^18^,^20^. Complete sequencing of the six IncI1/ST16 plasmids revealed that they consisted of a similar large backbone with considerable homology to the pEK204 plasmid, except for 8 regions missing in some of the plasmids compared to the reference, suggesting a possible similar origin.

Furthermore, five pairs of the IncI1/ST16 alleles in the present study(repI1, ardA, trbA, sogS and pill), are different with those of the IncI1/ST3 subtype, and four pairs of the IncI1/ST16 alleles (ardA, trbA, sogS and pill) are different to those of the IncI1/ST2 subtype, suggesting a lower genetic relationship between IncI1/ST16 and these two commonly described subtypes. Notably, the IncI2 (pHN1122–1) subtype plasmids that are commonly found in strains of animal origin in China and harbor *bla*_CTX-M-55_ were not detected in this study^13^. The IncI2 and IncI1 plasmids have an identical backbone gene encoding fimbriae that play an important role in engaging the plasmid and metastasis^21^. So far, the cause of high incidence of IncI1/ST16 carrying *bla*_CTX-M-55_ from the ESBL isolates remains unclear, and the relationship between IncI1/ST16 and other ESBL genotypes in clinical and animal-derived strains remains to be further studied.

Another significant plasmid subtype carrying the *bla*_CTX-M-55_ in our study was the IncF that accounted for 22%(10/45) of all *bla*_CTX-M-55_ carrying plasmids. IncF plasmids harboring *bla*_CTX-M-15_ are frequently detected among *Enterobacteriaceae* worldwide, and the acquisition of these plasmids is known to contribute to the dissemination of antimicrobial resistance and virulence genes^22^. There are currently 35 subtypes of IncF reported in plasmid MLST database. In the present study, we detected the IncF subtype F18: A-: B1, which is characterized by iron uptake (missing *eitABCD*), haemagglutinin and serum survival gene, and was firstly reported in the Avian Pathogenic *E. coli* (APEC)^23^. Other studies from Tunisia and China also reported high prevalence of the F18: A-: B1 subtype in *E. coli* strains of animal origin, and harbored the *oqxAB* or *bla*_CTX-M-1_ resistance genes^24^,^25^. These findings again suggest the possibility of horizontal transfer of the F18: A-: B1 plasmids between humans and animals.

An important feature of the ESBL-carrying plasmids is the existence of insertion fragments such as IS*Ecp1*, IS*26* and ISCR in these plasmids. For example, the IS*Ecp1* gene, often located at the 48 bp upstream of the *bla*_CTX-M-55_ gene, can be used to determine the homology of different CTX-M-ESBL genes^26^. Furthermore, the IS*Ecp1* gene was reported to be responsible for the mobility and expression of the *bla*_CTX-M_ gene. Qu and colleagues reported that the insertion of IS*Ecp1* gene at 45 bp, 48 bp and 127 bp upstream of the *bla*_CTX-M-55_ gene, induces the expression of this gene in plasmids^27^. In this study, 94% of IncI1 plasmids contained the IS*Ecp1* or the IS*26* insertion at 45 bp or 48 bp upstream of the *bla*_CTX-M-55_ gene, which suggests that acquisition of the *bla*_CTX-M-55_ gene by the Inc I1 plasmids might be mediated by the insertion fragments.

There are several limitations in our study. Firstly, only UTI ESBL-EC strains were analyzed; it is unclear whether the *bla*_CTX-M-55_ gene carrying ESBL-EC strains from other specimens such as blood belong to ST1193 and have IncI1 plasmids. Secondly, due to limited time and financial resources, a limited number (58 of 111, 52%) of isolates were used in conjugation studies and hence in plasmid analysis, which introduces some bias to our study. It’s arguable that the untested isolates would have yielded different results. Thirdly, to the best of our knowledge, this is the first report on the molecular epidemiology of the *bla*_CTX-M-55_-carrying major clone ST1193 and epidemic plasmid IncI1F/ST16 and F18: A-: B1. A whole genome analysis would greatly facilitate our understanding of the prevalence of the ST1193 and epidemic plasmid IncI1F/ST16 and F18: A-: B1 among the ESBL-EC strains. And finally, although *bla*_CTX-M-55_-carrying strains have been isolated from animal samples, there is insufficient evidence to conclude that there is dissemination of the *bla*_CTX-M-55_ gene between humans and animals.

In conclusion, the high prevalence of the *bla*_CTX-M-55_ gene among *E. coli* isolates from UTI patients was mainly attributed to the clonal dissemination of ST1193 strains, carrying the Inc I1/ST16 plasmids and mobile genetic element in China. This information can facilitate monitoring and controlling dissemination of the *bla*_CTX-M-55_ gene carrying strains so as to limit the rise in antibiotic resistance, which is a public health concern.

## Materials and Methods

### Ethics statement

This study protocol was approved by the Ethics Committee of The First Affiliated Hospital of Guangzhou Medical University. All subjects signed written informed consent prior to the study. Patient information was anonymized and de-identified prior to analysis. All methods were carried out in accordance with relevant guidelines and regulations that are stated in the methods below.

### Bacterial isolate collection

In our previous study, 137 out of 660 clinical ESBL-EC isolates from cases of UTI were shown to harbor *bla*_CTX-M-55_ gene. Of these137 isolates, only those that harbored a single *bla*_CTX-M-55_ gene (n = 111), were included in the current study. The isolates were collected from 21 general hospitals from 19 cities in China. These hospitals are distributed in seven geographic regions across China: four in Eastern China, two in Southern China, five in Northern China, two in Central China, two in Northeast China, two in Southwest, and four in Northwest. Clinical information of patients from whom the isolates were obtained, was reviewed retrospectively, including age, gender, underlying diseases, site and type of infection. All the bacterial isolates were re-identified using a VITEK2 Compact automated microbial identification system (bioMérieux, France). *E. coli* ATCC 25922 was used as the control strain.

### Antimicrobial susceptibility

The minimum inhibitory concentrations (MICs) of common antibiotics (National Institutes for Food and Drug Control, Beijing, China) against ESBL-producing *E. coli* were determined using the broth microdilution method with *E. coli* ATCC 25922 as the control strain. Interpretation of results followed the criteria of CLSI 2013^28^. The interpretation breakpoint of cefoperazone/Sulbactam (Pfizer Inc., USA) was extrapolated from that of cefoperazone, and those of tigecycline and colistin (Pfizer Inc.) were based on the criteria of the European Committee on Antimicrobial Susceptibility Testing (EUCAST-2012).

### Pulsed-field gel electrophoresis (PFGE)

PFGE analysis was conducted on the 111 isolates following a standardized protocol^29^, with electrophoresis performed using a CHEF DRIII System (Bio-Rad). The results were analysed by BioNumerics software (Applied Maths, St-Martens-Latern, Belgium) using the Dice similarity coefficient.

### Multilocus Sequence Typing (MLST)

MLST was carried out as previously described^30^. Different sequences of a given locus were assigned an allele number based on *E. coli* MLST database (http://mlst.warwick.ac.uk/mlst/dbs/Ecoli). Sequence types (STs) were obtained via the electronic database at the above website. The allelic profiles and defined clonal complexes (CCs) of all STs were further analyzed with the *B*ased *u*pon *R*elated *S*equence *T*ypes (BURST) clustering algorithm (eburst.mlst.net).

### Conjugation experiments and plasmid analysis

Conjugation experiments were performed on 16 of 20 ST1193 isolates, and a random selection (n = 42), of other isolates, making sure that at least one to three isolates from each hospital was included. Transferability of *bla*_CTX-M-55_ genes was determined by conjugation experiments using rifampin -resistant *E. coli* C600 as the recipient strain as described previously^31^. Transconjugants were selected on Mueller-Hinton agar plates containing rifampin (300 mg/L) and ceftazidime (2 mg/L). Incompatibility (Inc) groups, antibiotic resistance genes, and genetic environment were detected as described previously^32^,^33^. The sizes of plasmids of transconjugants were determined using S1-treated genomic DNA followed by PFGE (S1-PFGE). The epidemiological relatedness of IncI and IncF plasmids was determined by MLST and referred to the database (http://pubmlst.org/plasmid/)^23^,^34^.

### Complete nucleotide sequencing of the epidemic plasmids

A random selection of the same ST Inc I1 plasmids (n = 6) was sequenced by next generation sequencing. Total DNA (includes genome and plasmid) from the transconjugants was sequenced using Illumina MiSeq 300 PE platform.The sequence reads were de novo assembled using CLC genomics workbench version 8.0 (Qiagen).

## Additional Information

**How to cite this article:** Xia, L. *et al*. Prevalence of ST1193 clone and IncI1/ST16 plasmid in *E-coli* isolates carrying *bla*_CTX-M-55_ gene from urinary tract infections patients in China. *Sci. Rep.* **7**, 44866; doi: 10.1038/srep44866 (2017).

**Publisher's note:** Springer Nature remains neutral with regard to jurisdictional claims in published maps and institutional affiliations.

## Supplementary Material

Supplementary Information

## Figures and Tables

**Table 1:** Characteristics of plasmids in transconjugants analysed in this study.

Plasmid	pMLST(Kp)/RST^a^	Isolate	Region	ST	Other antibiotic resistance genes	Insertion fragments	Intergenic spacer region(bp)^b^
IncI1	ST16(97)	AH1	east	1193	*TEM-1*	IS*Ecp1*	48
	ST16(97)	AH14	east	12	*DHA*	IS*Ecp1*	48
	ST16(97)	AH30	east	1193	*TEM-1*	IS*Ecp1*	48
	ST16(97)	SD28	east	998	—	IS*Ecp1*	48
	ST15	SD33	east	167	*TEM-1, aac(3*′)*-II*	IS*26*	45
	ST16(97)	Z1-8	east	10	*TEM-1,qnrS*	IS*Ecp1*	48
	ST23	Z1-40	east	4208	*CMY-2*	IS*26*	45
	ST16(97)	ZS45	east	1193	*TEM-1, aac(3*′)*-II, qnrA*	IS*Ecp1*	48
	ST16(97)	GP20	south	44	*aac-(6*′)*-Ib*	IS*26*	48
	ST136	HN40	south	410	*TEM-1, aac(3*′)*-II, aac-(6*′)*-Ib,VEB,*	IS*Ecp1*	48
	ST16(97)	HN47	south	1193	*TEM-1*	IS*Ecp1*	48
	ST16(97)	HB41	north	1193	*TEM-1, aac(3*′)*-II*	ND^d^	ND
	ST16(97)	HB65	north	95	*TEM-1, aac(3*′)*-II,VEB*	IS*26*	45
	NT^c^	SX3	north	2003	*aac(3*′)*-II*	IS*Ecp1*	48
	NT	SX4	north	1193	—	IS*26*	48
	NT	TZ17	north	4204	—	IS*Ecp1*	48
	ST16(97)	TZ48	north	1193	—	ND	ND
	ST16(97)	XA41	north	10	*TEM-1, aac(3*′)*-II*	IS*Ecp1*	48
	ST16(97)	TJ4	central	1193	*TEM-1, aac(3*′)*-II*	IS*26*	45
	NT	TJ24	central	648	*TEM-1,aac(3*′)*-II,DHA*	IS*Ecp1*	48
	ST136	ZZ7	central	156	*TEM-1, aac(3*′)*-II,qnrA,*	IS*Ecp1*	45
	ST16(97)	ZZ25	central	131	*TEM-1,aac(3*′)*-II,aac-(6*′)*-Ib*	IS*Ecp1*	48
	NT	ZZ39	central	405	*aac(3*′)*-II*	IS*Ecp1*	48
	ST16(97)	1502	southwest	1193	*TEM-1, aac(3*′)*-II*	IS*Ecp1*	48
	ST16(97)	1642	southwest	1193	*TEM-1*	IS*26*	45
	ST16(97)	NX18	northwest	95	—	IS*Ecp1*	48
	ST16(97)	NX32	northwest	167	*TEM-1, aac(3*′)*-II, qnrA, qnrB*	IS*26*	48
	ST16(97)	HZ2	northwest	1193	*TEM-1*	IS*Ecp1*	48
	ST16(97)	QH11	northwest	1193	*TEM-1*	IS*26*	48
	ST16(97)	QR29	northwest	1193	*TEM-1, ARM*	IS*26*	45
	ST16(97)	JL13	northeast	648	*TEM-1, aac(3*′)(*3*′)*-II*	IS*Ecp1*	48
	ST16(97)	JL38	northeast	1193	—	IS*Ecp1*	48
	NT	SJ13	northeast	167	*aac(3*′)*-II*	IS*26*	48
	NT	SJ22	northeast	38	*TEM-1,aac(3*′)*-II*	IS*Ecp1*	48
IncF	F18:A-:B1 (145)	Z1-26	east	156	*aac(3*′)*-II*	IS*Ecp1*	48
	F18:A-:B1 (145)	ZS13	east	617	*aac(3*′)*-II*	IS*Ecp1*	48
	FII,FIA,FIB (NT)	HN34	south	405	*aac(3*′)*-II, qnrA,OXA-1,ARM*	IS*Ecp1*	48
	F-:A1:B1	HB24	north	1193	*qnrA*	IS*Ecp1*	45
	FII,FIA(NT)	ZR36	north	46	*qnrA*	IS*Ecp1*	48
	F-:A-:B20	1660	southwest	131	*aac(3*′)*-II*	IS*Ecp1*	48
	F18:A-:B1 (145)	SC19	southwest	354	*TEM-1*	IS*Ecp1*	48
	F18:A-:B1 (145)	SC34	southwest	156	*aac(3*′)*-II,*	IS*Ecp1*	48
	FII,FIA(NT)	JL25	northeast	405	*TEM-1, aac(3*′)*-II,*	IS*26*	45
	F51:A-:B10	SJ38	northeast	1193	—	IS*Ecp1*	48
IncP		NX45	northwest	95	*TEM-1, aac(3*′)*-II, IMP*	IS*Ecp1*	48

^a^Plasmids were identified in transconjugants. The allele number of the IncI1 pMLST profile (repI, ardA, trbA, sogS and pill) is indicated. NT, not typed. ^a^Plasmids were identified in transconjugants. Plasmid subtypes were classified according to their plasmidic MLST (pMLST) for IncI1 plasmids or to their FAB (FII, FIA, and FIB) formulas for IncF plasmids.

^b^Intergenic spacer region between insertion fragments and *bla*_CTX-M-55_.

^c^NT, not typed.

^d^ND, not detected.
