# Supplementary material for: Prevalence of ST1193 clone and IncI1/ST16 plasmid in E-coli isolates carrying blaCTX-M-55 gene from urinary tract infections patients in China
Source: Sci Rep. 2017 Mar 24;7:44866. doi: 10.1038/srep44866 (PMC5364490; doi:10.1038/srep44866)
Supplement: Supplementary Information [file srep44866-s1.pdf]

Prevalence of ST1193 clone and IncI1/ST16 plasmid in E-coli isolates carrying blaCTX-M-55 gene from urinary tract infections patients in China

Liang Xia[2] Ph.D.; Yang Liu[1] Ph.D.; Shu Xia[1]Ph.D.; Timothy Kudinha[3]Ph.D.; Shu-nian Xiao[1] M.B.; Nan-shan Zhong[1], M.D., FCCP; Guo-sheng Ren[2#] M.D.; Chao Zhuo[1#] M.D. FCCP

Table S1. Susceptibility of 111 CTX-M-55-producing Es*cherichia coli* isolates from UTIs patients against common antimicrobial agents

**Agent**	**Resistant(%)**	**Susceptible(%)**	**MIC50(ug/ml)**	**MIC90(ug/ml)**
Imipenem	2	98	0.125	0.25
Meropenem	0	100	<=0.06	<=0.06
Ertapenem	2	98	<=0.06	0.25
Tigecycline	6	94	0.25	0.5
Colistin	0	100	1	2
Cefoperazone/sulbactam	9.6	76.2	8	16
Piperacillin/ tazobactam	5	93	2	4
Cefepime	98	2	>32	>32
Cefotaxime	94	6	>64	>64
Ceftazidime	97	3	32	>64
Ceftriaxone	97.4	2.6	>64	>64
Aztreonam	97	3	>8	>8

Amikacin	18	82	4	>128
Levofloxacin	76	16	16	>16
Ciprofloxacin	85	15	>16	>16
Minocycline	26	58	4	>16

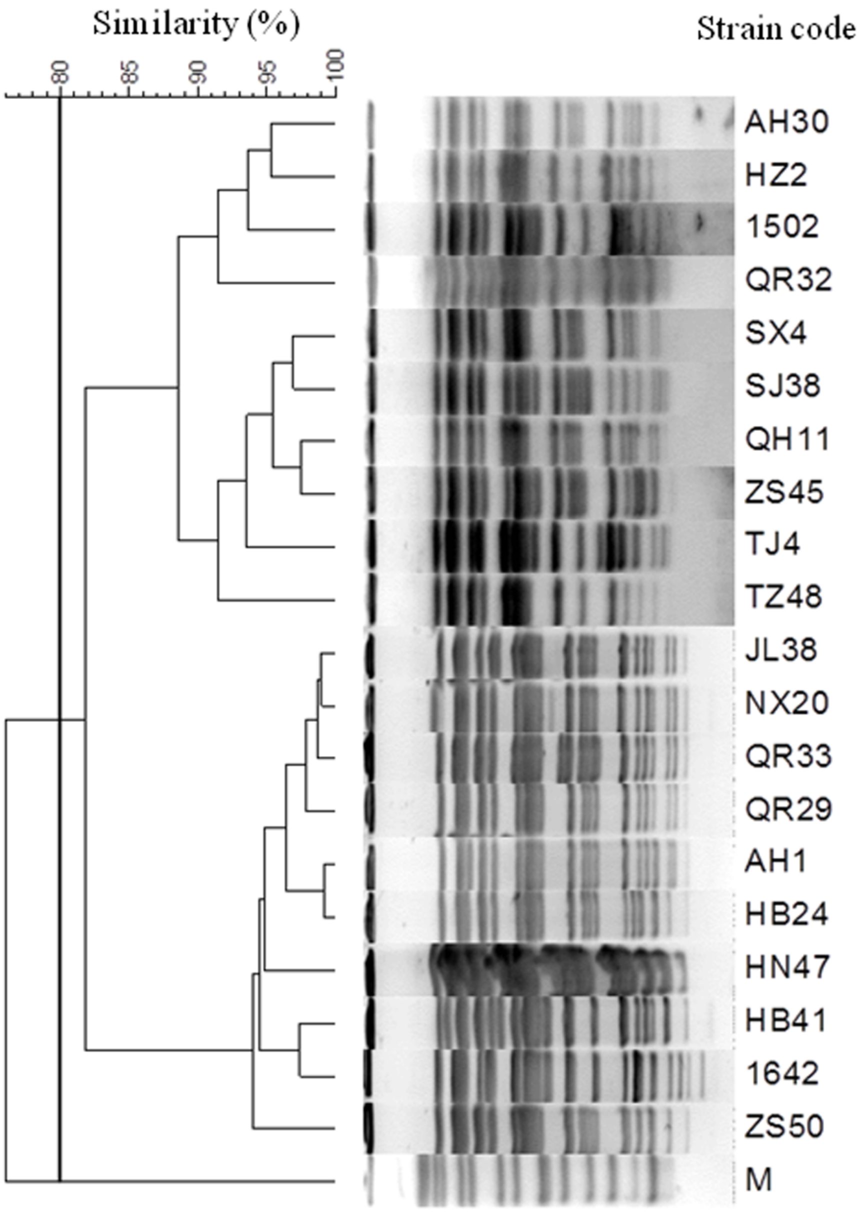

Figure S1 Dendrogram showing the relationship of Cluster Ⅰ. PFGE profiles were generated by XbaI digestion of genomic DNA. The thick black line indicates 80% banding pattern similarity, used to define strains.

Table S2. Distribution of sequence types(ST) among 111 *E.coli* UTIs isolates carrying *bla*$_{\mathbf{CTX\text{-}M\text{-}55}}$ gene

ST	Number(%) of isolates
ST1193	20(18.0)
ST405	9(8.1)
ST156	5(4.5)
ST2003	5(4.5)
ST12	4(3.6)
ST95	4(3.6)
ST167	4(3.6)
ST354	4(3.6)
ST10	3(2.7)
ST38	3(2.7)
ST101	3(2.7)
ST131	3(2.7)
ST4207[a]	1(0.9)
ST4208[a]	1(0.9)
ST4210[a]	1(0.9)
Various others[b]	
Untypable	5(4.5)

[a] ST4207,4208 and ST4210 were novel to this study

[b] Various others: A total of 36 STs occurring in one or 2 isolates each

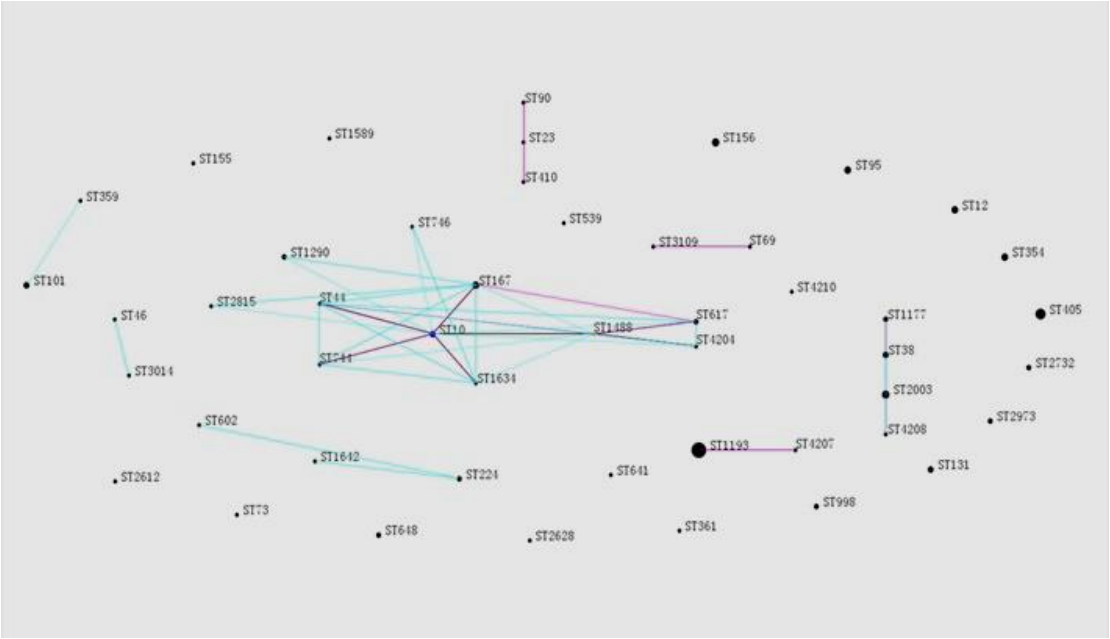

Figure S2 eBURST diagram of ST from isolates in the present study. Each ST is represented by a dot, the size of the dots represents the relative frequency of the ST. Predicted founders are positioned centrally in each cluster and shown in blue, SLVs and DLVs are shown in purple and green, respectively.

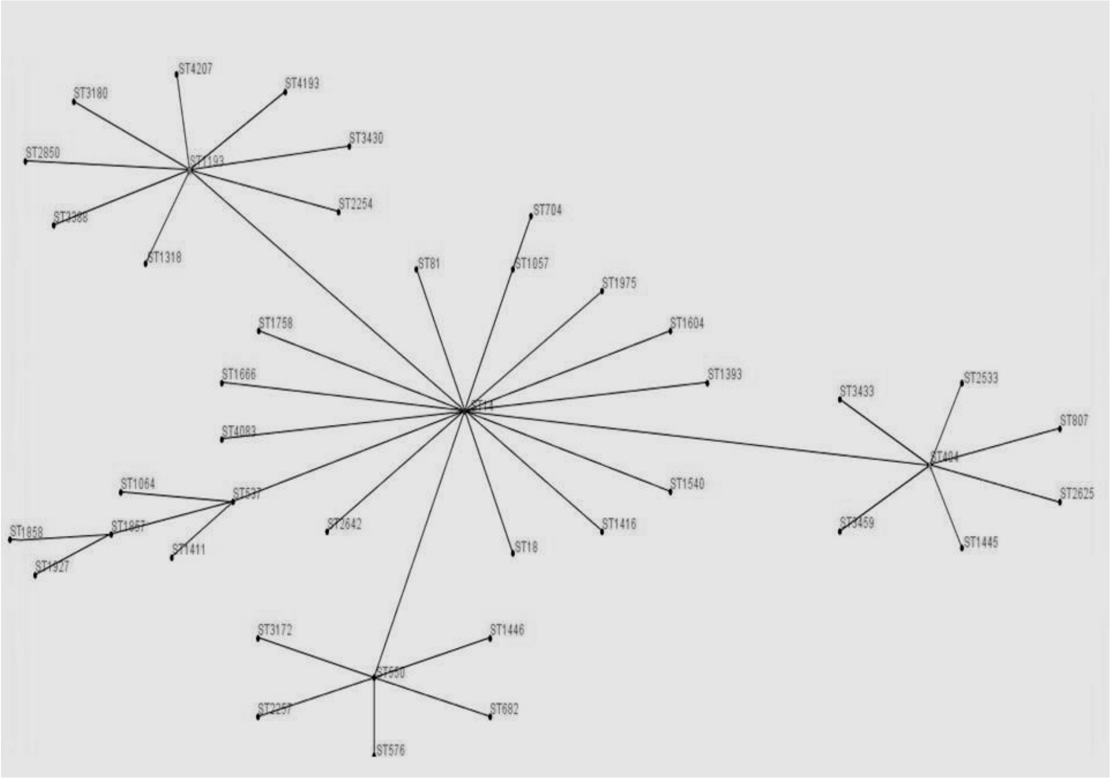

Figure S3 A snapshot of CC14 is shown in an eBURST comparison between the current dataset and all of the information held in the international online database.
